# Anti-amphiphysin antibody positive autoimmune syndrome: case series and literature review

**DOI:** 10.3389/fimmu.2026.1782004

**Published:** 2026-04-01

**Authors:** Linran Wang, Jingjiong Chen, Xin Tang, Haojun Yu, Dandan Guo, Xiaowei Chen, Li Cao, Kaili Lu

**Affiliations:** 1 Department of Neurology, Shanghai Sixth People’s Hospital Affiliated to Shanghai Jiao Tong University School of Medicine, Shanghai, China; 2 Suzhou Hospital of Anhui Medical University, Suzhou Municipal Hospital of Anhui Province, Suzhou, China; 3 Department of Genetics and Rare Diseases, Shanghai Sixth People’s Hospital Affiliated to Shanghai Jiao Tong University School of Medicine, Shanghai, China; 4 Shanghai Neurological Rare Disease Biobank and Precision Diagnostic Technical Service Platform, Shanghai, China; 5 Neurological Disorder Center, Haikou Orthopedic and Diabetes Hospital of Shanghai Sixth People’s Hospital, Haikou, China; 6 Shanghai Deji Hospital, Qingdao University, Qingdao, China

**Keywords:** amphiphysin antibody, auto-immunity, immunotherapy, myelitis, paraneoplastic neurological syndrome

## Abstract

**Background and purpose:**

Paraneoplastic neurological syndromes (PNS) is an uncommon disorder affecting the central nervous system (CNS), peripheral nervous system, or autonomic nervous system. The aim of this study was to analyze the clinical characteristics of anti-amphiphysin-IgG-positive autoimmune syndrome and to assess its association with a spectrum of​ anti-amphiphysin-IgG-associated autoimmune syndromes and tumors.

**Methods:**

We reported clinical data of four patients with anti-amphiphysin-IgG-positive autoimmune syndrome, and conducted a literature review.

**Result:**

Four amphiphysin-IgG-positive cases were identified. Within our cohort, antibodies feature was comprised of isolated amphiphysin-IgG (2/4), coexisting aquaporin-4-immunoglobulin G (AQP4-IgG) (1/4), coexisting N-methyl- D -aspartate receptor immunoglobulin G (NMDAR-IgG) (1/4). Clinical presentation included conus medullaris-cauda equina syndrome (1/4), myelitis (2/4) and stiff-person syndrome (SPS) (1/4). The coexisting cancer was involved of lung cancer (1/4).

**Conclusion:**

Anti-amphiphysin-IgG-positive autoimmune syndrome demonstrate heterogeneous clinical presentations, including myelitis, SPS, and rare variants like conus medullaris-cauda equina syndrome. Coexistence with other neural antibodies (e.g., AQP4-IgG or NMDAR-IgG) may elevate tumor risk. While strongly associated with malignancies such as breast and small cell lung cancer (SCLC), combined immunotherapy and oncologic therapy can stabilize or improve neurological function in subsets of patients. Early serologic testing and sustained cancer surveillance are imperative for prompt intervention and prognostic optimization.

## Introduction

Paraneoplastic neurological syndromes (PNS) is typically triggered by immune response against ectopically expressed neuronal antigens (1). It has been indicated that approximately 1 in 10,000 cancer patients are affected by PNS (2). A prospective cohort study involving 60,000 patients with suspected PNS revealed that 553 cases (0.9%) were tested positive for PNS-related antibodies (3). Currently, antibodies related to PNS are classified into two groups based on the location of the target antigen. One group targets intracellular neuronal antibodies, including anti-Hu, anti-Yo, anti-Ri, and anti-amphiphysin. The other targets neuronal surface antigens including anti-voltage-gated calcium channels (anti-VGCC) and anti-N-methyl- D -aspartate receptor (anti-NMDAR) (4). Furthermore, antibodies targeting astrocytic surface antigens, including anti-aquaporin-4 (anti-AQP4), have also been identified in paraneoplastic contexts, often associated with lung cancer and breast cancer (5). Amphiphysin is an intracellular protein essential for clathrin-mediated synaptic vesicle endocytosis (6). Unlike classical paraneoplastic antigens, it is sometimes classified as an intermediate antigen because it becomes transiently accessible to the extracellular space during vesicle fusion (7, 8). This transient exposure may allow autoantibodies to bind and exert pathogenic effects, a pathogenic mechanism that has been validated through passive transfer animal models (6). The anti-amphiphysin antibody was initially identified in patients with stiff-person syndrome (SPS) with breast cancer (9). Patients with anti-amphiphysin antibody positive PNS coexistence central nervous system (CNS) often present with SPS, but may also show encephalitis, myelitis, and peripheral neuropathy. The anti-amphiphysin antibody is considered a high-risk phenotype within PNS, and tumors are detected in over 70% of cases (4), mostly small cell lung cancer (SCLC) and breast cancer (10). In the majority of cases (76%), neuropathy precedes the cancer diagnosis (11). Early detection of antibodies is crucial for predicting and diagnosing tumors.

We reported four cases with anti-amphiphysin antibody positive. Amphiphysin-IgG was identified in serum or cerebrospinal fluid (CSF) samples at different concentration. Following immunotherapy, 2/4 of cohort marked improvement in neurological symptoms.

Additionally, this study summarizes a review of 47 English-language cases of anti-amphiphysin-IgG-associated autoimmune syndromes documented in PubMed from 1996 to 2024 utilizing the search terms (“Amphiphysin antibody” OR “Anti-Amphiphysin” OR “Amphiphysin IgG” AND “Paraneoplastic neurological syndrome” OR “Paraneoplastic syndrome” OR “Paraneoplastic neuropathy” OR “Stiff-person syndrome” AND “Case report” OR “Case series”). Through systematic screening of related articles, critical information had been extracted and tabulated. We reviewed 122 publications, from which 34 studies were included ultimately.

## Patients and methods

### Participants

We conducted a retrospective cohort study at a single center. The clinical data collected from patients hospitalized at Shanghai Sixth People’s Hospital Affiliated to Shanghai Jiao Tong University School of Medicine from January 2023 to May 2025. Patients were selected based on​ the following criteria: (i) Diagnosis of probable or definite anti-amphiphysin-IgG-associated autoimmune syndromes (4); (ii) Age ≥ 18 years old; (iii) Positive serum and/or CSF amphiphysin-IgG and other autoimmune antibodies confirmed by cell-based assay (CBA) and/or tissue-based assay (TBA) (AQP4-IgG, NMDAR-IgG, Hu, Ri, CV2, Ma1, Ma2, SOX1, Tr, Zic4, Titin, Recoverin, PKCγ, Yo, GAD65); (iv) Exclusion of HIV infection, B12 and folate deficiency, alcoholism and other autoimmune and inflammatory disorders.

### Clinical data collection

The core data elements were comprised of the following key variables: gender, age, clinical manifestations, laboratory findings, spine cord MRI results, therapeutic interventions, and clinical outcomes. All spine MRI examinations were performed on​ 3 Tesla (3T) scanners. Long-term outcomes were assessed during outpatient follow-up visits after discharge.

## Results

### Case 1

A 69-year-old female who was admitted for back pain, low limb numbness and weakness, along with urinary and fecal incontinence. Neurological examination revealed 4/5 strength in the bilateral lower extremities. Additionally, there was sensory loss in the saddle area and bilaterally reduced patellar and ankle reflexes. Lumbar spine MRI revealed mild swelling of the conus medullaris at the T12-L1 level with focal punctate enhancement. Brain MRI was unremarkable. The CSF analysis revealed a normal pressure (95 mmH_2_O), elevated protein levels (1.14 g/L) and normal white blood cell count (5*10^6/L). Metagenomic next-generation sequencing (mNGS) of the CSF was negative. Screening for antibody to amphiphysin was positive at 1:10 in serum and 1:1 in CSF but negative by TBA, while demyelinating antibodies (myelin oligodendrocyte glycoprotein, AQP4) and IgG-oligoclonal bands (OCB) were negative. Cancer screening through lung computed tomography (CT), breast ultrasound, and breast MRI revealed no noticeable abnormalities. Despite clinical recommendation for whole body positron emission tomography/computed tomography (PET/CT), however, the patient refused PET/CT examination due to financial constraints. Based on the symptoms, neurological examination and lumbar spine MRI, conus medullaris-cauda equina syndrome was diagnosed. Thus, the patient was diagnosed with conus medullaris-cauda equina syndrome associated with anti-amphiphysin antibody, which was categorized as probable PNS. Treatment with high-dose methyprednisolone achieved clinical remission. The patient remained clinically stable during the one-month follow-up but succumbed to acute myocardial infarction three months after discharge.

### Case 2

A 40-year-old man complained of bilateral lower limb numbness and weakness. Physical examination revealed strength was 4/5 in the left lower extremity proximally, 3/5 in the right proximally and 2/5 distally. Pinprick sensation, proprioception, and vibration were diminished below T10 bilaterally, but there was no sensory level or saddle anesthesia. Patellar reflex was normal in left, and hyperreflexia in right. Babinski sign was normal in left, and positive in right. MRA of the thoracolumbar and cervicothoracic revealed no abnormality. MRI of the cervical and thoracic confirmed C7-T1 and T6–9 inflammatory or demyelinating myelitis. Brain MRI was unremarkable. The CSF revealed normal pressure (90 mmH_2_O), elevated protein levels (0.51 g/L) and normal white blood cell count (3*10^6/L). The mNGS of the CSF was negative. Screening for antibodies to the amphiphysin proved positive at 1:32 in serum and at 1:1 in CSF, while the TBA was negative. Thus, he was diagnosed with myelitis associated with anti-amphiphysin. PET/CT revealed unremarkable. Six months after methylprednisolone therapy, follow-up MRI showed resolution of inflammatory lesions.

### Case 3

A 68-year-old man presented with subcostal pain accompanied by numbness in both lower limbs and urinary/fecal incontinence in 2023. The patient had a history of lung adenocarcinoma treated with right lobectomy in 2000, and subsequent radiotherapy after recurrence detected in 2022. Physical examination revealed muscle strength was 4/5 in both lower limbs and the remaining muscle strength was 5/5 in upper limbs. Pinprick sensation was diminished below the right costal arch. Vibration sense was reduced bilaterally below the anterior superior iliac spines. Bilateral Babinski signs were positive. The CSF revealed a normal pressure (120 mmH2O), normal protein levels (0.29 g/L) and normal white blood cell count (4*10^6/L). CSF mNGS was negative. The results of screening for antibodies to AQP4 at 1:32 and anti-amphiphysin at 1:30 were both positive in serum but negative in CSF, while the TBA was negative. The CSF showed type IV oligoclonal band. The thoracic spine MRI demonstrated a longitudinally enhancing T2 hyperintense lesion at T5-7. The cervical MRI, brain MRI, and thoracolumbar MRA revealed unremarkable. The unavailability of tumor specimens precluded immunohistochemical validation of antigen expression. The diagnosis was myelitis associated with neuromyelitis optica spectrum disorder (NMOSD) or probable PNS (4). Following treatment with corticosteroids and intravenous immunoglobulin (IVIG), his clinical symptom improved partially. Three months after the onset of the disease, a follow-up head MRI in the outpatient department showed high T2 signals with edema in the left frontal lobe, periventricular area, basal ganglia, and corpus callosum. Lung cancer with intracranial metastasis was considered. Four months after disease onset, the patient died due to intracranial metastasis of lung cancer accompanied by severe intracranial hemorrhage.

### Case 4

A 62-year-old woman with a history of cerebral infarction developed with herpes zoster, and after 7 days she walked unsteadily and accompanied by stiffness in both lower limbs. The patient did not experienced headache and seizure. On examination, hypertonia was observed in both lower extremities but muscle tone of other limbs was normal. The muscle strength of four limbs was normal. The CSF revealed normal pressure (115 mmH_2_O), elevated protein levels (0.50 g/L) and normal white blood cell count (2*10^6/L). CSF mNGS was negative. Laboratory workup revealed anti-NMDAR IgG was positive at 1:100 in serum and 1:1 in CSF, and serum anti-amphiphysin antibody was positive at 1:32, while the TBA was negative. Electroencephalogram showed no sharp-waves. Thoracic spine MRI and lumbosacral plexus MRI revealed no significant abnormalities. The brain MRI revealed no abnormalities. Gynecological ultrasound revealed no teratoma. She was diagnosed with SPS associated with probable anti-amphiphysin PNS. Following treatment with corticosteroids and efgartigimod, the patient returned to independent walking.

This study summarizes a review of 47 English-language cases of anti-amphiphysin-IgG-associated autoimmune syndromes documented in PubMed from 1996 to 2024. Among the 47 patients, there were 19 males (40.4%) and 28 females (59.6%), resulting in a male to female ratio of 1:1.47. The median age was 61.7 years (range, 25–85 years). For the 47 patients, primary clinical manifestations (**Table 1**) were SPS (n = 17), peripheral neuropathy (n = 10), limbic encephalitis (LE) (n = 6), encephalomyelitis (n = 2), Lambert-Eaton myasthenic syndrome (LEMS) (n = 3), brain stem encephalitis (n = 5), myelitis (n = 1), reversible cerebral atrophy (n = 1), cerebellar degeneration (n = 1), and undetermined clinical syndromes (characterized by reduced consciousness) (n = 1). Thirteen patients demonstrated occurrences of two or three symptoms.

**Table 1:** Syndromes and antibodies in 47 patients.

Syndrome	Number of patients	Additional information	Antibody	References (for literature cases)
SPS	17	Myelitis (2)	anti-GAD65 (1)	(24–40)
		LE (1)	anti-VGCC (1)	
		Peripheral neuropathy (1)		
		LE+Brainstem encephalitis+Myelitis (1) SPS+LE+LEMS (1)		
Peripheral neuropathy	10	Encephalomyelitis (1)		(38, 39, 41–46)
LE	6	Peripheral neuropathy (1)	anti-mitochondrial+anti-Hu (1)	(17, 41, 45, 47–49)
			anti-Hu (1)	
Encephalomyelitis	2			(45, 50)
LEMS	3	PCD (1)	anti-mitochondrial+anti-VGCC (1)、anti-VGCC (1)	(41, 45)
Brainstem encephalitis/ Rhombencephalitis	5	LEMS (1)	anti-VGCC (1)	(41, 44, 51–53)
Myelitis	1	Peripheral neuropathy (1)		(54)
reversible cerebral atrophy	1	Peripheral neuropathy (1)		(55)
PCD	1	Peripheral neuropathy (1)		(41)
Undefined	1			(56)

SPS, stiff-person syndrome; LE, limbic encephalitis; LEMS, Lambert-Eaton myasthenic syndrome; PCD, paraneoplastic cerebellar degeneration; anti-GAD65, anti-glutamic acid decarboxylase 65; anti-VGCC, anti-voltage-gated calcium channels.

40 (85.1%) cases were isolated amphiphysin-IgG, and 7 patients were positive in both amphiphysin-IgG and other antibodies. Among them, 5 patients were coexisted with one additional antibody (anti-VGCC, n = 3; anti-glutamic acid decarboxylase 65 (anti-GAD65), n = 1; or anti-Hu, n = 1), and 2 patients were combined two additional autoantibodies (mitochondrial-IgG and Hu-IgG, n = 1; mitochondrial-IgG and VGCC-IgG, n = 1). The tumor characteristics of 47 patients are summarized in **Table 2**. Breast cancer (n = 20, 42.6%) emerged as the predominant cancer in this review, followed by SCLC (n = 13, 27.7%), non-small cell lung cancer (NSCLC) (n = 2, 4.3%), appendiceal goblet cell carcinoma (n = 1), gastric adenocarcinoma (n = 1), lung adenocarcinoma (n = 1), intracranial diffuse large B-cell lymphoma (DLBCL) (n = 1), urinary bladder cancer (n = 1), prostate cancer (n = 1), and gynecologic cancer (n = 1). Five cases presented with unidentified neoplastic classification eventually. Among these, two cases demonstrated persistent absence of malignancy during follow-up, with the maximum follow-up duration reached 13 years. Another patient expired prior to completion of additional diagnostic procedures.

**Table 2:** Cancer subtypes.

Cancer	Number of patients
SCLC	13
Breast cancer	20
NSCLC	2
appendiceal goblet cell carcinoma	1
gastric adenocarcinoma	1
lung adenocarcinoma	1
primary central nervous system lymphoma	1
Urinary bladder cancer	1
Prostate cancer	1
ovarian carcinoma	1
NA	5

SCLC, small cell lung cancer; NSCLC, non-small cell lung cancer.

The treatment details of 33 patients were presented in **Table 3** (excluding 14 cases with unspecified therapy). Among them, 5 patients received only immunotherapy (including corticosteroids and IVIG) without improvement and 3 patients received only cancer treatment with alleviation in 2 patients. In addition, 24 patients received both therapies with improvement in 16 patients, and 1 received symptomatic treatment without improvement. Among these treated patients, 18 cases demonstrated clinical improvement (54.5%), while 15 cases showed no significant improvement (45.5%). Of the 11 patients who passed away during the study, 5 (45.5%) had identified evidence including cardiac arrest (n=1), lung cancer (n=2), and sepsis (n=2).

**Table 3:** Treatment and treatment response.

Therapy	Number of patients	Number of responders/all treateda
Immunotherapyb	5	0
Cancer therapyc	3	1/3
Both modalities	24	15/24
expectant treatment	1	
No therapy	14	
Total number of responders	18	
Total number of non-responders	15	
No response information or therapy	14	

a Stabilization and/or improvement of neurological symptoms in numbers of patients receiving each treatment modality.

b Corticosteroids, immunoglobulin or plasmapheresis, alone or in combinations.

c Surgery, hormone therapy or radiotherapy, alone or in combinations.

## Discussion

Our cohort include 4 cases with anti-amphiphysin-IgG-associated autoimmune syndromes, with 2 males (50%) and 2 females (50%), equating to ratio of 1:1. Our cohort’s median age (59.75 years) aligns with the review and larger series (12) (61.7 and 64 years, respectively), confirming the typical mid-to-later adulthood onset of amphiphysin-IgG syndromes. A notable divergence exists in sex distribution. Our cohort had a 1:1 ratio, contrasting with the female predominance (ratios of 1:1.47 and 1:1.52, respectively) in the review and larger series (12). This discrepancy may be attributed to our small sample size (n=4).

Patients with amphiphysin-IgG antibodies typically present with subacute or chronic onset. CNS-predominant phenotypes include SPS and encephalomyelitis, and peripheral nervous system manifestations is comprised of sensory neuronopathy​ and polyradiculoneuropathy. In our cohort, two myelitis cases demonstrated acute or subacute onset, while one presented with SPS and another with conus medullaris-cauda equina syndrome. The systematic study showed that SPS (n = 17, 36.1%) is most common CNS manifestation, confirmed by the research of Dubey et al (11). To our knowledge, there were no reports of conus medullaris-cauda equina syndrome caused by anti-amphiphysin antibody. It is important to note that CNS involvement may obscure the localization of peripheral nerve injury, thus nerve conduction studies and needle electromyographic play a crucial role in identifying peripheral neuropathy. Therefore, the patient extended the neurological syndromes spectrum in association with anti-amphiphysin antibody.

Within our cases, positive antibodies included isolated amphiphysin-IgG (2/4), coexisting AQP4-IgG (1/4) and coexisting NMDAR-IgG (1/4). The review study supports the high prevalence of isolated amphiphysin-IgG. Among those with more than one antibody, anti-VGCC, anti-GAD65 and anti-Hu antibody may occur, which could be accompanied by additional autoantibodies mitochondrial-IgG. Results from Dubey et al. showed that 63.6% were solely positive with amphiphysin-IgG, while 36.4% presented with additional antibodies, including Hu-IgG, CRMP5-IgG, or both (11). To the best of our knowledge, patient 3 was the first case positive for not only anti-amphiphysin antibody but also AQP4 antibody. He was diagnosed with paraneoplastic NMOSD associated with lung adenocarcinoma, though the pathogenicity of the amphiphysin antibody cannot be ruled out. Based on the diagnostic criteria advocated by the PNS-Care panel in 2021 (4), our patient was identified as “probable PNS”. A previous report showed that rare paraneoplastic AQP4-IgG-positive NMOSD patients were mostly older presenting with longitudinally extensive transverse myelitis (LETM) (5) and the predominant tumor was lung cancer and breast cancer (5, 13), which was consistent with our case. Tumor antigen release and cell death may be responsible for the presence of both anti-amphiphysin and anti-AQP4 antibody. In addition, patient 4 was the firstly reported patient positive for both anti-amphiphysin antibody and anti-NMDAR antibody. The clinical features of anti-NMDAR encephalitis included psychosis and epilepsy but not SPS, so we considered that the NMDAR antibody might merely be a bystander antibody (14).

The discrepancy between negative TBA and positive CBA in our cohort is consistent with recent findings regarding the limitations of rat brain immunohistochemistry (IHC). Nagata et al. (15) reported that while TBA is a valuable screening tool, it may miss up to one-third of antibody-positive cases, particularly when antibody titers are low. Their research highlighted that low titers (e.g., < 1:32) significantly decrease the detection rate of the “neuropil pattern” on commercial IHC. Additionally, technical factors in tissue preparation may affect the preservation of native epitopes, making certain antigens harder to detect via TBA than via CBA, which utilizes specific transfected cells to ensure high antigen accessibility.

The anti-amphiphysin antibody is recognized as a high-risk phenotype in PNS, primarily associated with underlying malignancies (4). In our retrospective study, we had one patient with lung cancer. In the systematic study, twenty-three cases (48.9%) were accompanied by malignant tumors, including breast cancer and SCLC. In our systematic study, among 6 patients with amphiphysin-IgG combined with other antibodies, all had tumors, including 5 cases of SCLC and 1 case of breast cancer. Prior studies by Pittock et al. and Qiao Lei et al. have demonstrated that the coexistence of amphiphysin-IgG with other autoantibodies is associated with a significantly increased rate of malignancies compared to patients displaying isolated amphiphysin-IgG (3, 16). The majority of patients (76%) experience neuropathic symptoms before their cancer diagnosis, typically four months in advance (11). The longest recorded duration without tumor detection was 13 years (17). Therefore, our patient’s tumor detection needs long-term monitoring. Active cancer screening is recommended in clinical practice for these patients to facilitate early detection and intervention. Generally, if the initial tumor screening yields negative results, it is recommended to repeat the screening every 3–6 months, followed by annual screenings for a total duration of 4 years (18).

In terms of treatment, first-line immunotherapy typically consists of corticosteroid, IVIG, or plasma exchange. If first-line interventions fail, second-line options include rituximab, cyclophosphamide, and mycophenolate mofetil. In our review, the majority of patients received immunotherapy combined with tumor therapy, with favorable outcomes. Therefore, anti-amphiphysin antibody-associated disorders may be more responsive to immunotherapy than disorders associated with typical intracellular antibodies such as anti-Hu or anti-Yo (6–8). Previous studies indicated that combination therapy is superior to monotherapy (11, 19). In our systematic review, the observed mortality rate was 45.5%. Previous studies have reported that the mortality rate among amphiphysin-IgG-positive patients ranges from 20% to 40%, because of cancer types, coexisting autoantibodies, and the treatment modalities implemented (11, 12, 19, 20). The most used treatment for anti-amphiphysin associated SPS was steroids, plasma exchange and oncologic therapy (21). In Case 4, efgartigimod was strategically administered as a targeted intervention following an inadequate response to initial corticosteroids and considering the high risk of IVIG associated with her history of cerebral ischemic stroke, which raised concerns regarding increased blood viscosity and thromboembolic events. As a neonatal Fc receptor (FcRn) antagonist, efgartigimod effectively enhances pathogenic IgG clearance through a mechanism that has been established as effective in other autoimmune neurological disorders such as myasthenia gravis and anti-GAD seropositive SPS (22). Our patient’s treatment combined with efgartigimod can quickly restore muscle strength, which was in line with the previous findings (23). Additionally, large prospective trials are indispensable for the longer period of efgartigimod in the management of anti-amphiphysin-IgG-associated autoimmune syndromes.

## Conclusion

This study consolidates the clinical spectrum of anti-amphiphysin-IgG-associated autoimmune syndromes, demonstrating that these disorders exhibit significant heterogeneity, ranging from myelitis and SPS to rare manifestations like conus medullaris-cauda equina syndrome. The coexistence of amphiphysin-IgG with other neural antibodies (e.g., AQP4-IgG or NMDAR-IgG) may amplify tumor risk and complicate clinical phenotypes, underscoring the necessity of comprehensive antibody profiling in high-risk patients. Although amphiphysin-IgG positivity is strongly associated with malignancies (particularly breast and lung cancers), combined immunotherapy and oncological management can stabilize or improve neurological outcomes in a subset of patients. Early antibody detection and sustained tumor surveillance remain critical for timely intervention.

Prospective multicenter studies are needed to validate updated diagnostic criteria and optimize treatment protocols for antibody-coexisting cases. Further investigations should elucidate the mechanisms underlying amphiphysin-IgG interactions with other autoantibodies and their synergistic effects on tumorigenesis. Additionally, exploring novel immunomodulatory approaches (e.g., targeted B-cell therapies or checkpoint inhibitor management) may address unmet needs in refractory cases. Long-term follow-up of seropositive patients without initial tumor detection will clarify the natural history and inform evidence-based surveillance strategies.

## Data Availability

The original contributions presented in the study are included in the article/**Supplementary Material**. Further inquiries can be directed to the corresponding authors.
