# Supplementary figures and images for: Anti-amphiphysin antibody positive autoimmune syndrome: case series and literature review
Source: Front Immunol. 2026 Apr 1;17:1782004. doi: 10.3389/fimmu.2026.1782004 (PMC13079297; doi:10.3389/fimmu.2026.1782004)

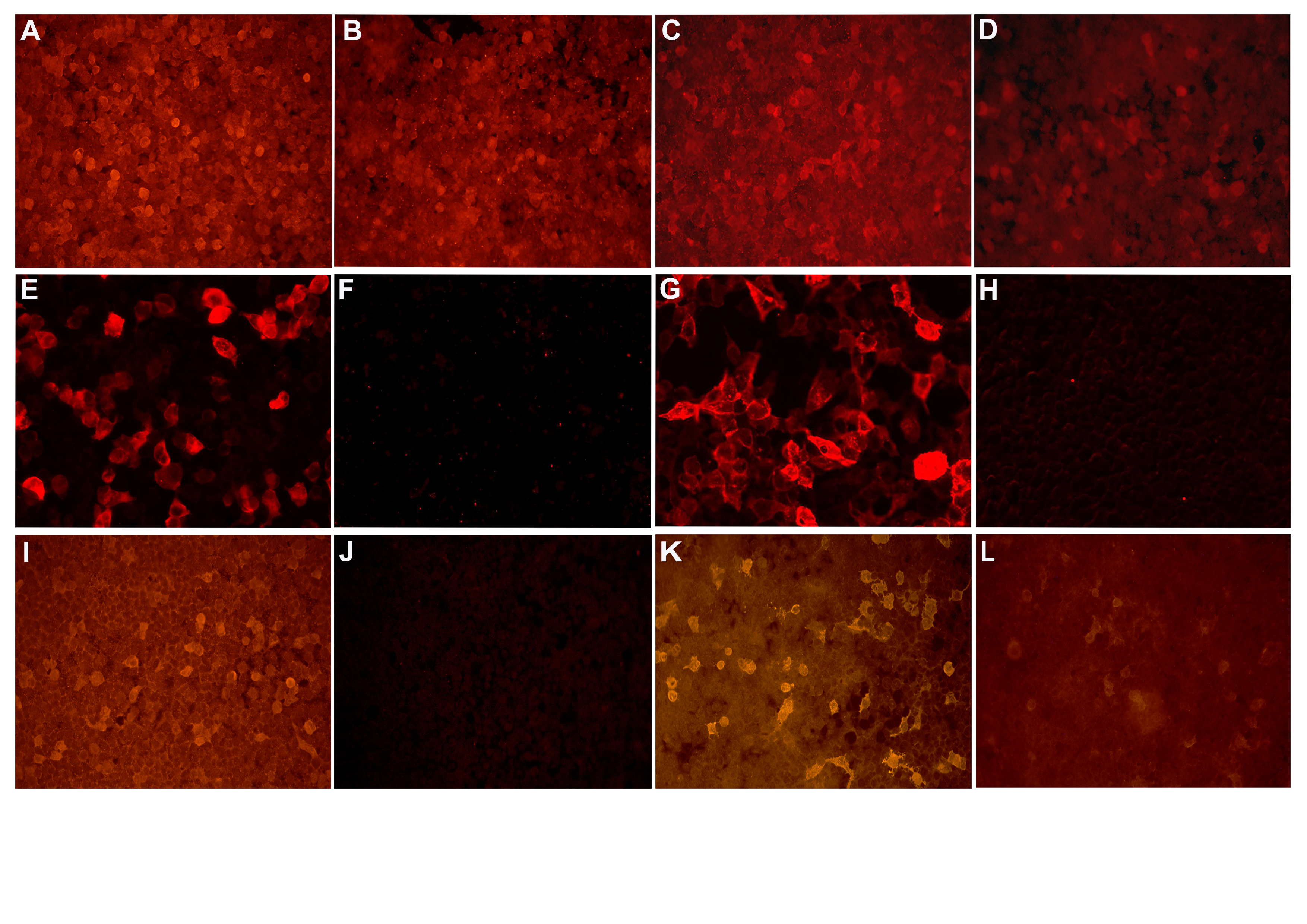

Supplement: Supplementary file 1 [file Image1.tif]
