# Supplementary material for: Anti-amphiphysin antibody positive autoimmune syndrome: case series and literature review
Source: Front Immunol. 2026 Apr 1;17:1782004. doi: 10.3389/fimmu.2026.1782004 (PMC13079297; doi:10.3389/fimmu.2026.1782004)
Supplement: Supplementary file 2 [file Table1.docx]

**Supplementary Figure 1. Immunofluorescence testing for neural autoantibodies using cell-based assay (CBA)**

CBA results are shown for all patients using 293T cells transiently transfected with human amphiphysin, AQP4, or NMDAR.

(A–B): Positive anti-amphiphysin IgG in serum (A) and CSF (B) by CBA in Patient 1.

(C–D) Positive anti-amphiphysin IgG in serum (C) and CSF (D) by CBA in Patient 2.

(E–H) Co-existence of autoantibodies in patient 3 with lung adenocarcinoma and myelitis. CBA showed positive anti-amphiphysin (E) and anti-AQP4 (G) IgG in serum, while both were negative in CSF (F and H, respectively).

(I–L) Overlapping autoimmunity in patient 4 presenting with stiff-person syndrome (SPS). CBA showed positive anti-amphiphysin IgG in serum (I) but negative in CSF (J), whereas anti-NMDAR IgG was detected in both serum (K) and CSF (L).
